# Corticomuscular and intermuscular coherence during evidence accumulation in sensorimotor decision‐making

**DOI:** 10.14814/phy2.70237

**Published:** 2025-03-18

**Authors:** Yvonne F. Visser, W. Pieter Medendorp, Luc P. J. Selen

**Affiliations:** ^1^ Donders Institute for Brain, Cognition and Behaviour Radboud University Nijmegen The Netherlands

**Keywords:** corticomuscular coherence, decision‐making, evidence accumulation, intermuscular coherence, motor control

## Abstract

Evidence accumulation processes during decision‐making are thought to continuously feed into the motor system, preparing multiple competing motor plans, of which one is executed when the evidence is complete. Previously, the state of this accumulation process has been studied by reading out the preparatory state of the motor system with evoked responses, once per trial. In this study, we aim to continuously track the sensorimotor decision during the trial using corticomuscular (CMC) and intermuscular coherence (IMC). We recorded EEG and EMG of healthy young adults (*n* = 34) who viewed random dot motion stimuli, with varying strengths across trials, and indicated their perceived motion direction by reaching towards one of two targets, requiring either flexion or extension of the elbow. Coherence was computed in the beta band. After stimulus presentation, both CMC and IMC show an initial phasic pattern, which is followed by sustained coherence patterns at a level that depends on stimulus strength for CMC. Prior to reach onset, the CMC for different stimulus strengths had a tendency to settle at similar levels. This tendency tentatively marks a stimulus‐independent decision bound. We conclude that CMC, and to a lesser extent IMC, track the evidence accumulation process on a single trial.

## 1 INTRODUCTION

There is increasing evidence that perception, decision making, and action do not reflect a serial neural chain but arise from overlapping and intertwined neural processes (Cisek & Kalaska, 2005; Contemori et al., 2023; de Lafuente et al., 2015; Donner et al., 2009; Gold & Shadlen, 2003; Rogge et al., 2022; Roitman & Shadlen, 2002; Selen et al., 2012, 2023; Visser et al., 2023; Yang et al., 2011). This means that the state of the decision‐making process, for example, about the direction of a saccade or a reach, is seen deeply within the motor system (Donner et al., 2009; Gold & Shadlen, 2003; Kelly & O'Connell, 2013), even in the periphery (Selen et al., 2012; Visser et al., 2023). Indeed, motor evoked potentials (Michelet et al., 2010), express visuomotor responses (Contemori et al., 2023), and stretch reflexes (Selen et al., 2012; Visser et al., 2023) all show characteristics of a decision making process. However, because these responses are evoked—during or at the end of a trial—they only provide a single estimate of the state of the motor system at a specific time point. Inferences about the temporal evolution of the peripheral motor system's state require multiple trials with evoked responses at different time points. Yet, tracking the state of the motor system on a single trial is critical to understand its link with ongoing evidence accumulation and convergence towards a decision.

To quantify this link, in the present study we use corticomuscular (CMC) and intermuscular (IMC) coherence. These measures can capture the changes in the descending drive to the motor periphery (Boonstra & Breakspear, 2012; Fries, 2005). CMC reflects coherent oscillations in motor cortex and muscle activity, which has been hypothesized as a mechanism to improve neuronal communication (Fries, 2005). CMC, which is typically found in the beta band, modulates not only with muscle load (Baker et al., 1997; Conway et al., 1995), but is also affected by movement requirements and online corrections (Kasuga et al., 2018; Kilner et al., 2000; Schoffelen et al., 2005; Spedden et al., 2022). Furthermore, CMC has been shown to covary with corticospinal excitability (van Elswijk et al., 2007), another measure associated with the central drive to the motor periphery. These findings suggest that motor preparation, and by extension decisions between different motor alternatives, is reflected in CMC. In support, Schoffelen et al. (2005) showed that the readiness to respond correlates with CMC. Likewise, Schoffelen et al. (2011) showed that a movement‐based report in a perceptual detection task is preceded by increased CMC.

Similar types of modulations of local beta band oscillations were reported in relation to changing task demands (Grent‐'t‐Jong et al., 2014; van Ede & Maris, 2013). In primary motor and dorsal premotor cortex, a build‐up in the lateralization of the beta band has been associated with upcoming perceptual responses (Donner et al., 2009). Beta‐band desynchronization prior to movement has also been investigated in relation to response uncertainty. For example, Doyle et al. (2005) manipulated the reliability of a cue indicating with which hand to respond and found increased desynchronization in the contralateral hemisphere prior to movement. Similarly, when manipulating the number of possible responses, Tzagarakis et al. (2010) reported more beta‐band desynchronization when fewer target options were presented. Lastly, beta band desynchronization was shown to be stronger for instructed versus free‐choice movements with either hand (van Helvert et al., 2021). These changes in cortical beta band power have clear temporal dynamics (Kilavik et al., 2013), making them likely to affect CMC measures over time as well. For long‐range synchronizations, van Wijk et al. (2009) showed modulation of lateralized beta‐band corticospinal coherence when selecting between competing motor responses, with lower contralateral coherence for high predictive cues.

IMC, reflecting the oscillatory coupling between two muscles and hypothesized to reflect their common synaptic input, is modulated by central drive (Boonstra et al., 2015; Farina et al., 2013). IMC has mainly been studied in the context of gait and posture (Boonstra et al., 2015; Jensen et al., 2019; Nojima et al., 2018; Sato & Choi, 2019; Spedden et al., 2022). For example, Sato and Choi (2019) show that early adaptation is associated with higher IMC, which returns to baseline after adaptation—supporting the idea that IMC increases with the amount of central control. Furthermore, when participants know a priori which limb to step with, IMC between bilateral muscles increases prior to the “go”‐cue, whereas IMC remained constant if the required stepping leg only became apparent at the ‘go’‐cue (Nojima et al., 2018). IMC between upper limb muscles has only been studied in relation to force production and fatigue (Danna‐Dos Santos et al., 2010; Farina et al., 2013). To our knowledge, the influence of movement readiness or available perceptual evidence on IMC has not been studied.

Here, we used CMC and IMC to track information flow to the peripheral motor system during the formation of a perceptual decision, expressed by a movement‐based report. Participants were instructed to indicate the direction of a random‐dot motion (RDM) stimulus, making an elbow flexion or extension movement, while we recorded EMG and EEG. If there is a continuous influence of accumulated sensory evidence on the preparatory state of the motor periphery, a time‐ and stimulus‐dependent increase of CMC and IMC is expected, reflecting the accumulation of sensory evidence and final convergence towards a decision.

We show that both beta‐band CMC and IMC increase with stimulus presentation and decrease prior to the initiation of the movement‐based report of the decision. Furthermore, CMC—but not IMC—shows a stronger phasic pattern for higher RDM stimulus strengths. However, CMC values for different stimulus strengths settle on a similar level right before reach onset. We conclude that CMC, and to a lesser extent IMC, track the ongoing accumulation of sensory evidence.

## 2 METHODS

### 2.1 Participants

Forty healthy, right‐handed participants took part in the experiment after providing written informed consent. Data of six of them were excluded due to technical issues with the recordings (3 participants) or lack of general task performance (3 participants). This left 34 participants for analysis (26 female, 8 male, age 23.6 ± 5.3 years). The local ethics committee of the Faculty of Social Sciences of Radboud University independently reviewed the study and had no formal objection.

### 2.2 Set‐up

Participants were seated in front of a robotic manipulandum (Howard et al., 2009), holding the handle with their right hand. The location of the handle was tracked with a 1000 Hz sampling rate. The forearm was supported by an airsled placed on the table below the handle, facilitating frictionless reaching movements in the horizontal plane. A mirror, mounted above the handle, reflected the display of a 27‐inch monitor (Asus MG279Q, 2560 × 1440 pixels, refresh rate 144 Hz) while blocking the arm from view. The monitor presented experimental information, including the target locations, the RDM stimulus, and the location of a cursor representing the hand position.

Electroencephalography (EEG) and electromyography (EMG) were recorded at 1000 Hz using BrainVision (BrainProducts, Munich, Germany). We measured 32‐channel EEG using actiCAP snap active electrodes, configured following the 10–20 system. The reference electrode was placed on the right mastoid, and for the ground electrode we used Poz instead of the typical ground location on the forehead to prevent interference from the monitor support close to the participants' forehead. Impedance was <25 kΩ in all electrodes included for the analysis. EMG was recorded using snap electrodes (Great Lakes Neuro Technologies Inc.), which were placed on the skin over the muscle belly of the biceps and triceps muscles. The reference electrode was placed on the end of the humerus. A photodiode placed over the screen detected the on‐ and offset of the RDM stimulus.

### 2.3 Paradigm

Participants were asked to make a perceptual decision about the direction of a random dot motion stimulus and report their decision by reaching, requiring an elbow flexion or extension movement, to one of two targets as soon as they made their decision.

**FIGURE 1:**
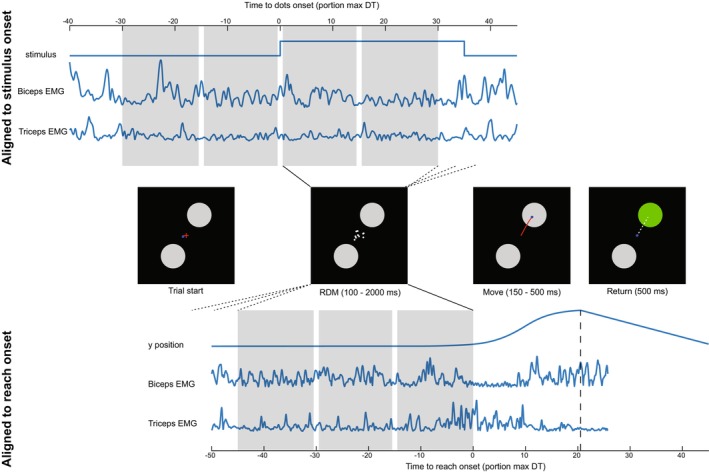
Experimental design. The middle row shows a schematic of a single trial. Participants had to keep their hand cursor (blue dot) on the central fixation cross (red). Next, the fixation cross was replaced by an RDM stimulus (the number of dots in this figure are schematic and do not reflect actual stimulus parameters). Participants had to reach to the target that matched the inferred direction of the stimulus. In this case, a reach towards the extension target is plotted, requiring triceps activation and relaxation of the biceps. Participants received feedback about their choice after the reach (target turning green or red), and the robotic manipulandum brought the hand back to the central location (white dashed line). The trajectory of the cursor was never visible to the participant and only plotted here, in red, for clarity. The upper row shows windowing for the stimulus‐aligned coherence analysis. Windows are expressed as a percentage of the longest decision time (DT) per participant. The first two windows occur before stimulus onset and the last two windows during the RDM presentation. In the lower row the windows are aligned to reach onset. All windows occur during the RDM presentation, before reach onset. Arrival in the chosen target is indicated with a vertical dashed line around 20% of normalized decision time.

A trial started when the hand cursor (1 cm diameter) was in the central start location (the “fixation cross” in Figure 1a, left panel). Two circular reach targets (diameter 10 cm, color white) appeared in opposite directions (55° and 235°, defined clockwise from the positive x‐axis) 10 cm away from the start location in the horizontal plane. Reaching for the bottom‐left target required elbow flexion while reaching to the top‐right target required elbow extension. After the hand had been stationary for 500 ms, the RDM stimulus, spanning 2.5° visual angle, was centrally displayed at one of five coherence levels: 0%, 3.2%, 6.4%, 12.8%, 25.6%, or 51.2%, with the dots moving either in the direction of the flexion or extension target. The stimulus consisted of 3 dot banks, resulting in a density of 15.6 dots/degree^2^/second and a dot movement speed of 7 degrees/second. For more details about the implementation of the RDM stimulus, see Newsome and Paré (1988) or Roitman and Shadlen (2002). To avoid confusion with corticomuscular coherence, we henceforth use *stimulus strength* to refer to the coherence level of the RDM stimulus. Participants were instructed to start a reach as soon as they had deciphered the stimulus direction. Once the cursor distance from the central start location exceeded 0.5 cm, the RDM stimulus disappeared. A maximum viewing duration of 2 s was allowed. At the end of the reach, the target turned green in case of a correct decision or red for an incorrect decision. When the stimulus had no direction (0% stimulus strength), correct or incorrect feedback was randomly determined. Participants continuously compensated for a 5 N force in the 55° direction—towards the extension target. This background load elicited a non‐zero baseline activity in the biceps muscle, enabling the detection of both task‐dependent increases and decreases in EMG and CMC in relation to this muscle.

Participants were asked to reach into the target, which felt mechanically as a soft cushion, without decelerating or instantiating an active return movement to minimize recruitment of the antagonist muscle. The cushion was simulated by a velocity‐dependent force for which the damping factor was ramped from 0 Ns/cm when entering the target to 0.75 Ns/cm in the center of the target. Once the handle was stationary, the robotic manipulandum brought it back to the starting position with a constant velocity of 10 cm/s. The next trial started 500 ms later. If no reach was started within 2 s or the handle did not become stationary inside one of the targets within 500 ms after reach onset, the trial resulted in an error and was repeated.

CMC and IMC analyses require the same number of trials for fair comparison of the different conditions. As a result, we aimed to have the same number of correctly performed trials (~150) for each stimulus strength. Based on the percentage correct trials for the different stimulus strengths observed during piloting, we tested 150 trials for both the 0% and 51.2% stimuli, 250 trials for the 3.2% and 6.4% stimuli, 200 trials for the 12.8% stimulus, and 174 trials for the 25.6% stimulus.

The number of RDM stimuli in the direction of the flexion and extension target was balanced for each stimulus level. Each participant performed 6 blocks of 196 trials (the last block contained 194), interleaved with short breaks (about 1 min, self‐paced by the participant). A practice block of around 100 trials—depending on performance—was done prior to the start of the experiment. During this block, participants were verbally encouraged to make their decision as fast as possible—always within 2 s—and to move towards the chosen target. They were explicitly instructed not to decelerate or to move back to the home position, letting the robotic manipulandum do this for them.

### 2.4 Data analysis

Data were analyzed in MATLAB. Electrophysiological data were preprocessed, segmented, and subjected to frequency and coherence analyses using the Fieldtrip toolbox (Oostenveld et al., 2011).

Decision time was defined as the time between the onset of the RDM stimulus and the initiation of the reach. Data from the robotic manipulandum were used to determine whether the correct target was selected. We chose to include only trials in which participants made the correct decision to avoid artificially inflating our effect for different RDM stimulus strengths. An incorrect decision is likely associated with a lower decision variable, and since the proportion of incorrect decisions differs between RDM stimulus strengths, we risk creating a false effect. Also see Figure 4b,c in Selen et al. (2012). For 0% coherence trials, neither response is (in)correct, and all trials were included.

#### 2.4.1 EEG/EMG preprocessing

EEG data were low‐pass filtered at 250 Hz, and a 50 Hz line‐noise filter was applied. Bad channels noted during the experiment were removed, after which the data was rereferenced to a common average, and an ICA was performed to remove blink artifacts. EMG data was rectified using the Hilbert transform and subsequently high‐pass filtered at 10 Hz. After filtering and artifact removal, both EEG and EMG data were cut into trial segments for 2 different alignments: relative to stimulus onset and relative to reach onset.

#### 2.4.2 Coherence analysis

Corticomuscular coherence (CMC) and intermuscular coherence (IMC) were computed for the different stimulus strengths and in several time windows relative to stimulus onset and reach onset. Windows were normalized according to the participant's longest decision time. Since there were large individual differences in participant's decision speed, the normalization aimed to ensure we were sampling similar stages of the decision process for each participant. For the stimulus onset aligned analysis we used 4 windows with relative decision times between −30% and 30%, whereas for the reach onset‐aligned analysis 3 windows with a relative decision time between −45% and 0% were used (see Figure 1 upper and lower row). The length of the windows ranged from 120 to 320 ms depending on the maximum decision time for each participant. These windows were chosen to reflect cortico‐muscular and inter‐muscular communication at the beginning (stimulus onset) and end (reach onset) of the decision‐making process, without contamination by kinematic changes in hand position. Given the dependency of the CMC and ICM measures on the S/N ratio (Bayraktaroglu et al., 2013), we stratified the trials such that we had an equal number of trials per time window and stimulus strength. The same trials were selected for each time window, and windows in which less than five trials were available for one of the stimulus strengths were excluded for that participant. The number of trials used for the analysis per participant can be found in Table S1.

We first computed the spectral power for each EEG and EMG channel between 10 and 80 Hz using multitaper frequency transformation resulting in bins of both 10.9 and 14.5 Hz. Then we computed both corticomuscular coherence (CMC) and intermuscular coherence (IMC) using the coherence method, which is described in detail in Schoffelen et al. (2011) and Rosenberg et al. (1989). CMC was computed between the activity recorded by 5 pre‐selected electrodes over the motor cortex (Cz, C3, C1, FCz, CP1) and the activity of the biceps. The same analysis was performed for the triceps. The resulting matrices, that is, coherence for the different time windows, frequency bins, and electrodes, were baselined by a time window just before the stimulus onset for both alignments. Subsequently, based on conventions in the literature (Baker, 2007; Dal Maso et al., 2017; Schoffelen et al., 2011), we averaged the CMC across the 5 electrodes and the frequency bins between 10 and 40 Hz (beta band). This resulted in a single CMC measure per stimulus strength and time window for each participant. Similarly, IMC was computed between the EMG signals of the biceps and triceps, baselined, and averaged between 10 and 40 Hz. This again resulted in one measure per stimulus strength and per time window for each participant.

Both the beta‐band CMC and IMC were z‐scored across all time windows and stimuli per participant. Then, the average coherence development over time was computed for all trials, and for different contrasts: (1) small versus large stimulus strengths, corresponding to low and high levels of evidence, (2) positive versus negative stimulus strengths, corresponding to extension, and flexion target reaches. The small versus large contrasts were investigated for flexion and extension evidence separately, while the positive versus negative contrasts were investigated for all stimulus strengths taken together.

#### 2.4.3 Statistical analysis

We performed a one‐way repeated‐measures ANOVA to investigate the effect of time on CMC and IMC values. To estimate the effect of stimulus strength, we used a 4 × 2 ANOVA in the stimulus‐aligned analysis and a 3 × 2 ANOVA in the reach‐aligned analysis. The factors taken in consideration were time window and stimulus strength, with participant as a random factor. Both main and interaction effects were allowed. Post‐hoc pairwise *t*‐tests were performed for individual contrasts following a significant main effect from the ANOVA. A Bonferroni correction was applied to adjust the significance level based on the number of comparisons.

## 3 RESULTS

**FIGURE 2:**
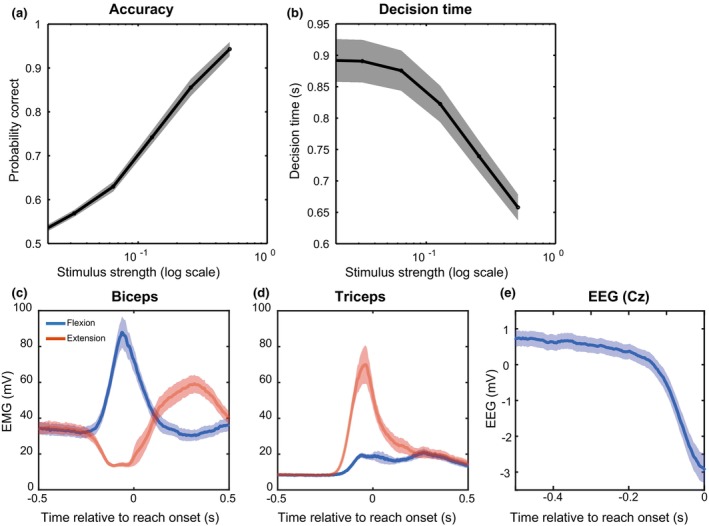
Behavioral performance, directional EMG activity, and readiness potential. (a) Grand average (*n* = 34) of accuracy of choices per stimulus strength. (b) Grand average of decision time per stimulus strength. (c) Grand average EMG response for the biceps when reaching for the flexion versus extension target. (d) Grand average EMG response for the triceps when reaching for the flexion versus the extension target. (e) Grand average preparatory activity (i.e., readiness potential) over the Cz electrode prior to reach onset. Shaded areas in all panels indicate S.E.M.

Participants viewed an RDM stimulus with different stimulus strengths and were asked to reach for the target corresponding to the perceived motion direction. Participants were sensitive to our experimental manipulation, showing an increase in the percentage of correct decisions with increasing stimulus strength (Figure 2a), while decision time decreased (Figure 2b).

Flexion and extension choices are clearly observable in the motor output, with biceps EMG activity increasing for the flexion target and decreasing for the extension target. This (de)activation starts about 250 ms before reach onset (Figure 2c). The triceps was engaged for reaches to the extension target and—to a much lesser degree—the flexion target (Figure 2d), also being activated about 250 ms before reach onset. Due to the background load, higher baseline activity is observed for the biceps (Figure 2c) compared to the triceps (Figure 2d). The EMG activity thus shows a distinct pattern for reaching to the two different targets.

The event‐related potential in EEG electrode Cz prior to reach onset shows a slow voltage decrease (Figure 2e). This voltage decrease over motor cortex is known as the readiness potential (RP), a widely recognized signature of motor preparation prior to voluntary action (Kornhuber & Deecke, 1965; Schurger et al., 2021).

In further analyses of CMC, we will focus on the biceps muscle only, as its activity can be up‐ and down‐regulated in preparation of the movement due to the background load, where the triceps muscle can only be upregulated (Figure 2c, van Ede & Maris (2013)). Results for the triceps are reported in Figures S1C and S2.

### 3.1 Spectral power of the CMC and IMC peaks in the beta band

**FIGURE 3:**
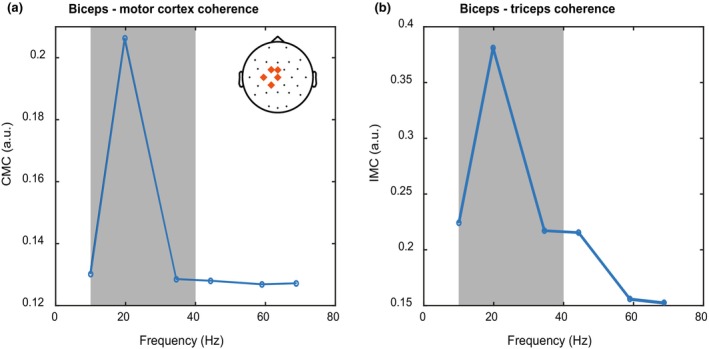
Power spectra of CMC and IMC. (a) Frequency spectrum of the corticomuscular coherence between biceps and the motor cortex near reach onset. (b) Frequency spectrum of the intermuscular coherence between biceps and triceps near reach onset. Gray areas indicate the part of the frequency spectrum that was included in the rest of the analysis for both panels. Note that the high‐pass filter applied to the EMG data prevents us from considering frequencies lower than 10 Hz.

We computed the CMC between the EEG potentials from Cz, C3, C1, FCz, and CP1 electrodes (covering the left motor cortex) and Hilbert‐transformed EMG recorded from the biceps. Figure 3a shows CMC as a function of frequency in the time window just before reach onset. In line with previous findings (Liu et al., 2019), there is a peak in the beta band, at 20 Hz. We focused on averaged CMC in frequencies between 10 and 40 Hz (gray area in Figure 3) for further analysis.

Intermuscular coherence was computed between Hilbert transformed biceps and triceps EMG. Like in the CMC analysis, a clear IMC peak was visible at 20 Hz in the time window just before reach onset.

### 3.2 CMC and IMC increase after stimulus onset and decrease before reach onset

**FIGURE 4:**
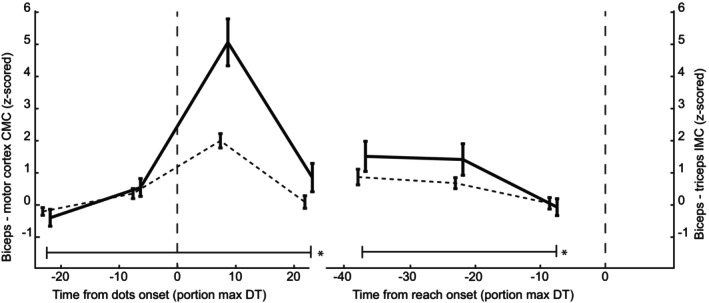
CMC development over time. Corticomuscular coherence (solid) and intermuscular coherence (dashed) relative to stimulus and reach onset (vertical dashed lines). All stimulus strengths were averaged together. Error bars represent S.E.M., and markers on the *x*‐axis are staggered for plotting purposes only.

Next, we analyzed the change in CMC within the 10–40 Hz band over time, both relative to stimulus onset and reach onset. Figure 4 illustrates the time‐resolved changes in the CMC and IMC across all trials (irrespective of stimulus strength and direction). While the CMC is relatively constant prior to stimulus onset, there is a small but significant increase between the first and second window (*t*(33) = 2.6, *p* = 0.018), perhaps due to anticipation of the stimulus. After stimulus onset, there is a phasic pattern of the CMC, which is followed by a decrease for the last time window (Figure 4, solid lines, left part). In the reach‐aligned analysis, a sustained pattern is visible, which then decreases towards the onset of the reach (Figure 4, solid line, right part). A 1‐way repeated measures ANOVA revealed a significant main effect of time on the stimulus‐onset aligned CMC, as well as on reach‐onset aligned CMC (*F*(34,3) = 27.02, *p* < 0.001 & *F*(34,2) = 4.12, *p* = 0.0195, respectively). The IMC shows a similar temporal pattern (dashed line in Figure 4). Again, the IMC increases slightly before stimulus onset (*t*(33) = 2.65, *p* = 0.012). The ANOVA revealed a main effect of time for the stimulus‐aligned analysis and the reach‐aligned analysis in IMC as well (Figure 4, dashed lines, *F*(34,3) = 30.9, *p* < 0.001, *F*(34,2) = 6.63, *p* = 0.002).

### 3.3 CMC is modulated by stimulus strength and converges before reach onset

**FIGURE 5:**
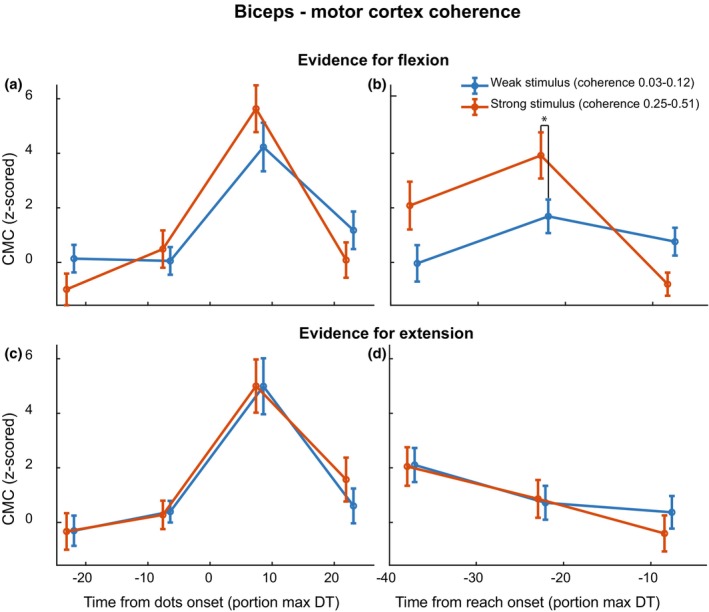
Corticomuscular coherence of the biceps as a function of time relative to stimulus onset (left column) and relative to reach onset (right column). The top row represents trials in which the stimulus direction was towards the lower left target (flexion) while the bottom row represents those in which the stimulus direction was towards the upper right target (extension). Trials with strong stimulus strength (25.6% and 51.2%) are averaged together and plotted in red, while trials with weak stimulus strength (3.2%, 6.4%, and 12.8%) are plotted in blue. Error bars represent S.E.M., and markers on the x‐axis are staggered for plotting purposes only.

If the CMC and IMC modulate with the dynamics of evidence accumulation, we expect that different stimulus strengths lead to different CMC and IMC dynamics. Since the biceps has a preferred direction to the flexion target, we expect this modulation to depend not only on stimulus strength but also on the direction of the inferred visual motion. We therefore analyzed the data according to a two‐by‐two design (Figure 5, stimulus strength (small: <=12.8%, large: > = 25.6%) × motion direction (flexion, extension)). After stimulus onset (Figure 5a,c), a phasic pattern occurs in the CMC, irrespective of stimulus strength. The decrease prior to reach onset (Figure 5b,d) is modulated by the stimulus strength, with higher levels of CMC for higher stimulus strengths (in orange) in the direction of the flexion target, which requires recruitment of the biceps muscle (Figure 5b). This modulation, however, was not significant (*F*(34,1) = 3.57, *p* = 0.066). The ANOVA also revealed an interaction effect between time and stimulus strength for both stimulus‐ and reach‐aligned CMC with flexion evidence (Figure 5b, *F*(34,2) = 12.26, *p* < 0.001). Post‐hoc pairwise *t*‐tests indicated an effect of stimulus strength at time 2 (*t*(33) = −2.524, *p* = 0.0166). In drift‐diffusion models, a fixed decision threshold is often assumed, which—when crossed—leads to movement initiation (e.g., see Roitman and Shadlen (2002)). Here, we see the difference between CMC for strong and weak stimuli disappear right before reach onset, which could be a signature of crossing this threshold (see Section 4).

**FIGURE 6:**
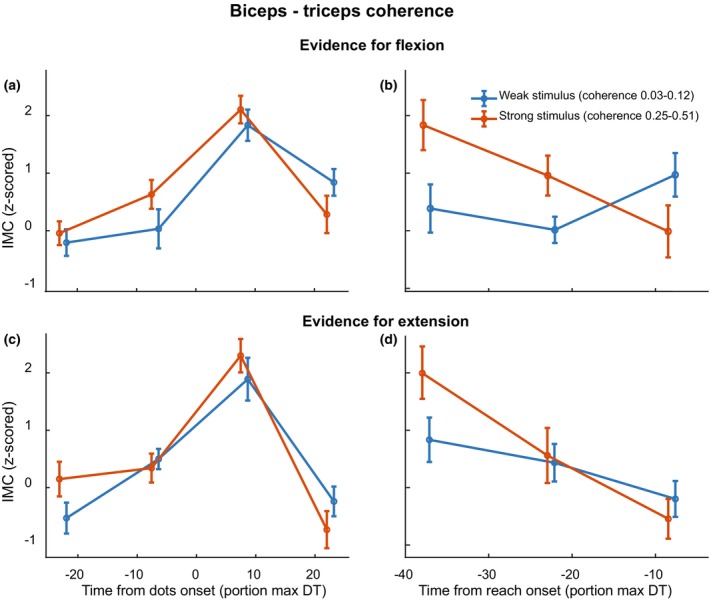
Intermuscular coherence as a function of time relative to stimulus onset (left column) and relative to reach onset (right column). The top row represents trials in which the stimulus direction was towards the lower left target (flexion) while the bottom row represents those in which the stimulus direction was towards the upper right target (extension). Trials with strong stimulus strength (25.6% and 51.2%) are averaged together and plotted in red, while trials with weak stimulus strength (3.2%, 6.4%, and 12.8%) are plotted in blue. Error bars represent S.E.M., and markers on the *x*‐axis are staggered for plotting purposes only.

We performed the same analyses on the IMC (Figure 6) to examine if the strength and direction of the stimulus affected the similarity between the activities of the two muscles. Though the temporal effect remained, no significant effect of stimulus strength was found on the IMC between biceps and triceps, either for evidence for the flexion or extension target (Figure 6, *p* > 0.05). However, an interaction effect between time and stimulus strength was found for the reach‐aligned IMC when evidence was in the flexion direction (Figure 6b, *F*(34,2) = 7.75, *p* = 0.001).

### 3.4 Time and stimulus direction interact for biceps CMC and IMC

**FIGURE 7:**
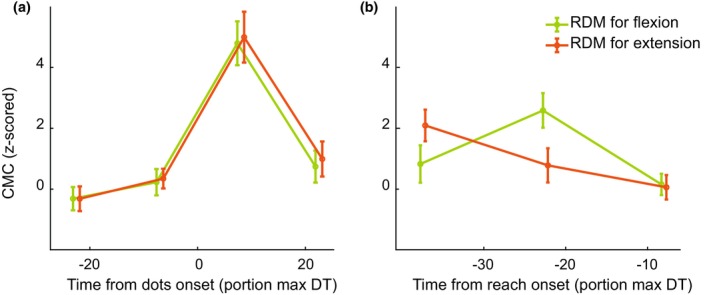
Corticomuscular coherence of the biceps for different stimulus directions. CMC was aligned to (a) stimulus onset or (b) reach onset. Trials in which the stimulus was in the direction of the flexion target were averaged together and plotted in green, while the average of all trials in which the stimulus was in the direction of the extension target is plotted in orange. Error bars represent S.E.M., and markers on the x‐axis are staggered for plotting purposes only. The same results for triceps CMC, as well as IMC can be found in Figure S1.

To strengthen our finding of convergence of CMC prior to reach onset, we also considered the effect of stimulus direction alone on preparatory CMC and IMC. To this end, we averaged all stimuli with evidence for the flexion target and all stimuli with evidence for the extension target separately. We found an interaction effect between time and stimulus direction for reach‐aligned CMC of the biceps (Figure 7b, *F*(34,2) = 5,05, *p* = 0.010) as well as stimulus‐aligned IMC (Figure S1B, *F*(34,3) = 6.17, *p* = 0.001). Since no main effect of stimulus direction was found, no post‐hoc *t*‐tests were run to find differences in CMC level for flexion and extension direction for specific time points. However, the interaction effect and visual inspection does suggest that the CMC values for flexion and extension evidence also converge prior to reach onset.

## 4 DISCUSSION

We investigated the continuous communication of decision‐related information from an RDM stimulus to the peripheral motor system using corticomuscular and intermuscular coherence (CMC/IMC). Participants responded faster and more accurately with increased stimulus strength and showed distinct muscle activation patterns for the two reach targets (Figure 2). In both CMC and IMC, a sharp increase was observed right after stimulus onset. Prior to reach onset, CMC and IMC displayed a negative slope (Figure 4). Moreover, confirming our hypothesis, we found that CMC values increased with increasing stimulus strength, mainly for the reach‐aligned analysis (Figure 5b). This finding suggests that neural correlates of the decision process can also be found in the interaction between motor cortex and the motor periphery. Additionally, we found that CMC for different stimulus strengths tend to settle at a similar level right before reach onset. The CMC values for different stimulus strengths, while separated for most of the trial, came to a similar value at reach onset, a possible signature of a stimulus‐independent decision bound. When considering the effect of stimulus direction, we observe a separation of the CMC values for flexion and extension, which we hypothesize to reflect differences in preparation for the two movement directions. This separation, like the one for stimulus strength, shows convergence to a similar value right before reach onset (Figure 7b).

Phasic pattern changes in CMC after stimulus onset have been observed before. For example, when participants are asked to report a stimulus change with a wrist flexion, CMC increases after stimulus onset which is readily followed by a rapid decrease (Schoffelen et al., 2011). Similarly, van Ede and Maris (2013) found a sustained increase in contralateral CMC in anticipation of a tactile stimulus serving as a ‘go’ cue followed by a decrease in beta band EMG once that stimulus occurred and a response had to be made. However, these studies did not investigate modulations of the CMC by the amount of available evidence, like we do in this paper.

We hypothesized that CMC would increase prior to reach onset, matching the increase in accumulated evidence. Instead, CMC shows a negative slope as it approaches reach onset, in each trial selection we performed (Figure 5b,d). According to the hypothesis by Fries (2005), where increased oscillatory coupling facilitates neural communication, this would mean a decrease in communication from the middle of the decision‐making process to the final execution of it. Though surprising, the observed decrease in coherence does match earlier observations (see above, Schoffelen et al., 2011; van Ede & Maris, 2013).

Alternatively, the observed decrease in CMC could be related to response uncertainty. Suppression of central beta band oscillations prior to a voluntary movement, called event‐related desynchronization (ERD), have been reliably shown in different paradigms. This ERD can be modulated by stimulus characteristics, such as response uncertainty (Kilavik et al., 2013; Toro et al., 1994). When a participant is more certain about the required response, a larger suppression is found (Doyle et al., 2005; Tzagarakis et al., 2010; van Helvert et al., 2021). In our paradigm, response uncertainty is higher for weaker stimulus strengths, when participants are making a more difficult perceptual decision. As a result, a similar desynchronization in the beta band may underly the pre‐reach reduction in CMC for strong stimulus strengths seen in Figure 5b. Though note that a direct relationship between changes in cortical beta power only and changes in CMC has previously been questioned (Baker & Baker, 2003). Our CMC findings likely reflects beta‐band suppression prior to voluntary movement, but might also be modulated by an increase in response uncertainty throughout the trial.

The approach of biceps CMC from trials with different stimulus strengths before reach onset mimics observations from the visual saccadic system. For example, lateral intraparietal cortex (LIP) firing rates corresponding to different reaction times converge to a common level prior to saccade onset (Churchland et al., 2008; Hanks et al., 2014). Also in an RDM stimulus decision‐making paradigm, firing rates in LIP differed between different stimulus strengths, but converged towards saccade onset (Roitman & Shadlen, 2002). According to popular drift diffusion models, the decision moment (and thus response onset) is marked by crossing a decision threshold (Ratcliff & McKoon, 2008). With differences in stimulus strength, the speed of evidence accumulation differs, but the final accumulated evidence needs to exceed the same threshold. Here, our results suggest a similar bound crossing in CMC: regardless of the strength of the stimulus, the CMC approaches a similar level when the participant makes their response.

The phasic pattern of the visual stimulus presentation on the CMC is in line with earlier findings. For example, Safri et al. (2006) showed that coherence between EEG and EMG in the beta band was increased when a visual stimulus was presented versus when isometric contractions were performed without visual information (Safri et al., 2006). More generally, task‐relevant visual information has been recovered from motor cortex activity alone, even if the required motor response is orthogonal to this visual information (Eisenberg et al., 2011; Shen & Alexander, 1997). Since our stimulus presentation was preceded by a black screen, the appearance of the visual stimulation has a widespread effect throughout the brain. Even in the IMC, which is based on signals not involved in visual processing whatsoever, we find an effect of stimulus presentation. The fact that stimulus presentation affects the IMC measure this drastically already supports our hypothesis that there is a continuous influence of decision‐relevant information on the peripheral motor system. However, the IMC and CMC pattern prior to reach onset could therefore also be explained simply by a temporal extinguishing of the initial response to the visual stimulus. Future studies might mitigate this problem by presenting continuous visual stimulation which contains shifts in direction, as in Kelly and O'Connell (2013).

## 5 CONCLUSION

In conclusion, we have shown a temporal corelate of decision‐making in the motor periphery. This temporal aspect is modulated by the amount of available evidence, furthering the idea of continuous influence of evidence between central motor areas and the periphery. Additionally, we found CMC signals stemming from different stimulus strengths settling on a similar level prior to reach onset, supporting bounded evidence accumulation.

## FUNDING INFORMATION

This work was supported by an internal grant from the Donders Centre for Cognition. W.P.M. is additionally supported by the following grants: NWA‐ORC‐1292.19.298, NWO‐SGW‐406.21.GO.009, and Interreg NWE‐RE:HOME.

## CONFLICT OF INTEREST STATEMENT

The authors declare no conflicts of interest.

## ETHICS STATEMENT

The study was performed in accordance with the declaration of Helsinki. Ethical approval was obtained from a local ethics committee (approval number ECSW‐2022‐083), and participants provided written informed consent before participating.

## Supporting information


Appendix S1.

## Data Availability

The data that support the findings of this study, as well as the analysis code used are openly available in the Radboud Data repository at https://data.ru.nl/collections/di/dcc/RDC_2022.00012_695.
