# Supplementary material for: Corticomuscular and intermuscular coherence during evidence accumulation in sensorimotor decision‐making
Source: Physiol Rep. 2025 Mar 18;13(6):e70237. doi: 10.14814/phy2.70237 (PMC11919635; doi:10.14814/phy2.70237)
Supplement: Supplementary file 1 — Appendix S1. [file PHY2-13-e70237-s001.docx]

**Supplementary materials**

**Supplementary table 1.** The number of trials per stimulus strength selected for analysis in each participant. Note that the maximum possible number of trials (if the participant responded correctly on each trial) would be 75 since we performed the analysis using signed stimulus strengths.

| **Number of trials per coherence** | | |
| --- | --- | --- |
| **Participant** | **Stimulus onset** | **Reach onset** |
| **1** | 36 | 36 |
| **3** | 42 | 18 |
| **4** | 33 | 33 |
| **5** | 63 | 63 |
| **6** | 58 | 7 |
| **7** | 54 | 54 |
| **9** | 67 | 15 |
| **10** | 58 | 37 |
| **11** | 67 | 18 |
| **14** | 43 | 6 |
| **16** | 52 | 9 |
| **17** | 52 | 52 |
| **18** | 59 | 59 |
| **19** | 49 | 49 |
| **20** | 23 | 23 |
| **21** | 65 | 10 |
| **22** | 63 | 61 |
| **23** | 57 | 12 |
| **24** | 64 | 43 |
| **25** | 60 | 11 |
| **26** | 33 | 33 |
| **27** | 59 | 20 |
| **28** | 33 | 33 |
| **29** | 25 | 25 |
| **30** | 51 | 13 |
| **31** | 60 | 27 |
| **32** | 65 | 13 |
| **33** | 58 | 58 |
| **34** | 60 | 5 |
| **35** | 39 | 39 |
| **37** | 47 | 47 |
| **38** | 61 | 18 |
| **39** | 52 | 52 |
| **40** | 52 | 31 |

**
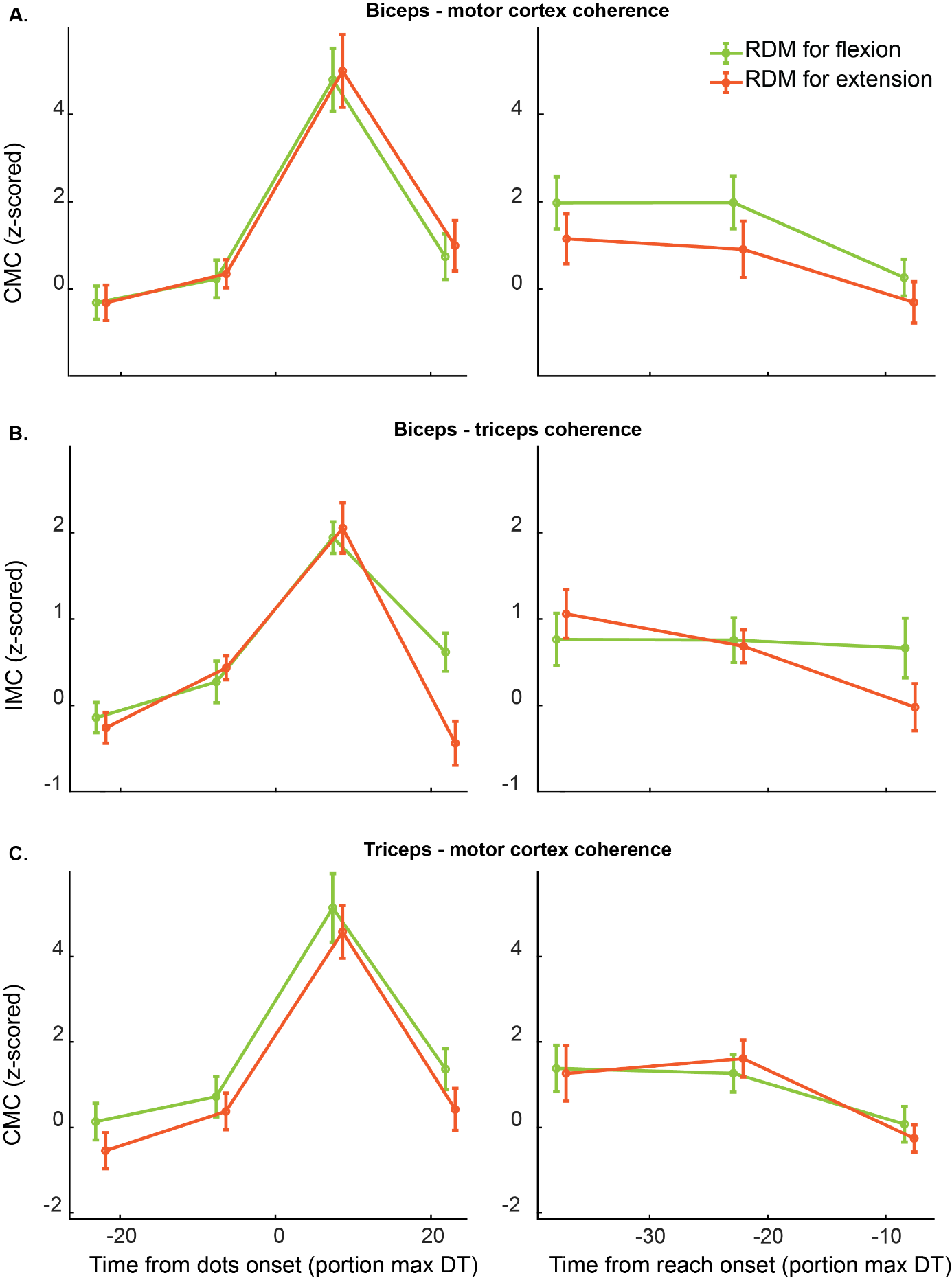
**

**Figure S1.** Corticomuscular/intermuscular coherence between **A)** biceps and motor cortex **B)** biceps and triceps and **C)** triceps and motor cortex. Left column shows development over time relative to dots onset, while the right column is relative to reach onset. Stimuli with different sign have been averaged together, indicating evidence for the different targets. Error bars represent S.E.M., and markers on the x-axis are staggered for plotting purposes only. A significant effect of dots direction was observed in the stimulus onset aligned analysis for triceps-motor cortex (panel C, F(34,1) = 4.84, p = 0.035).


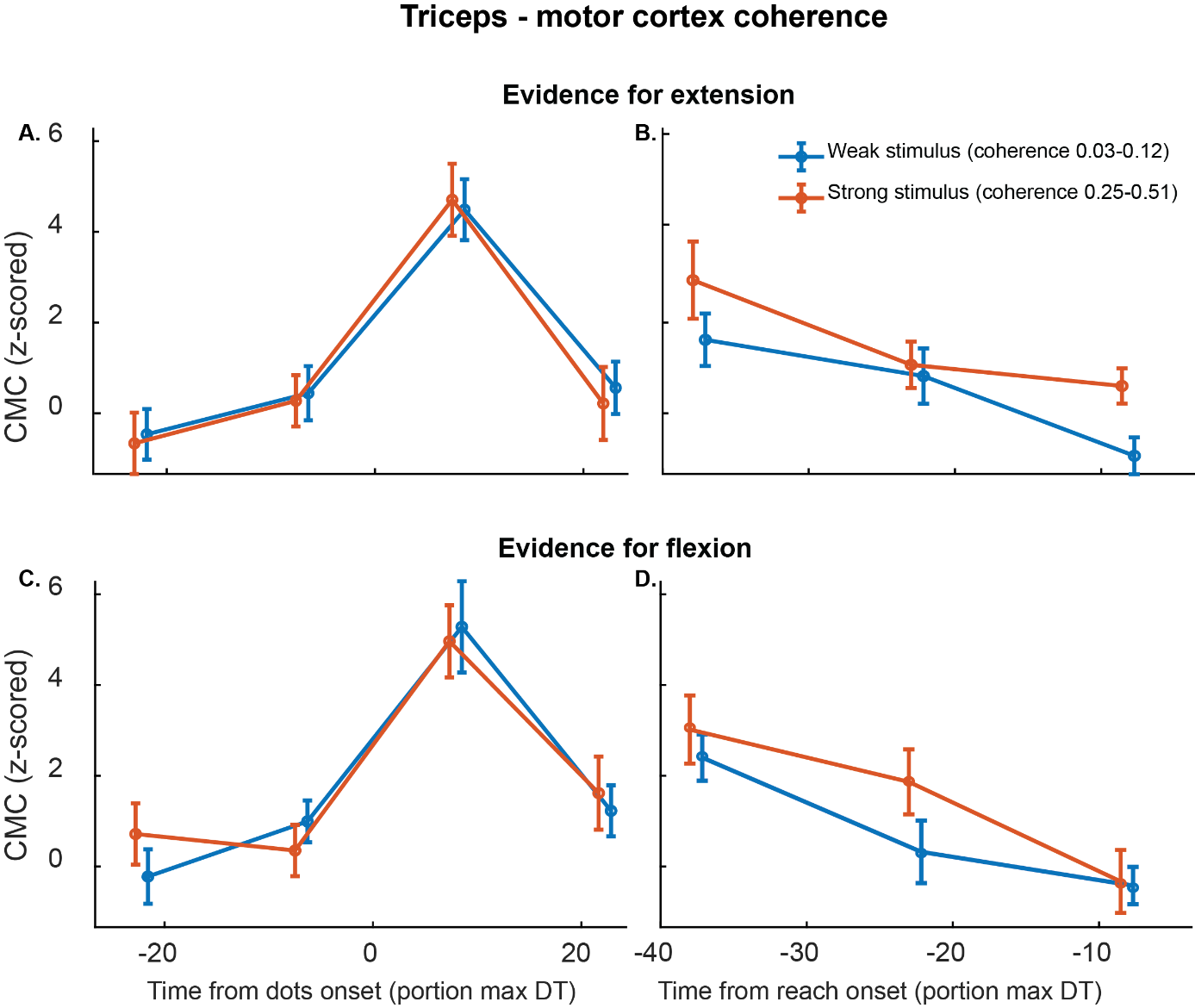


**Figure S2. Corticomuscular coherence of the triceps** as a function of time relative to stimulus onset (left column) and relative to reach onset (right column). The top row represents trials in which the stimulus direction was towards the extension target while the bottom row represents those in which the stimulus direction was towards the flexion target. Trials with strong stimulus strength (25.6% and 51.2%) are averaged together and plotted in red, while trials with weak stimulus strength (3.2%, 6.4% and 12.8%) are plotted in blue. Error bars represent S.E.M., and markers on the x-axis are staggered for plotting purposes only. A main effect of time was found for CMC with evidence in both directions and for alignments to both dots- and reach onset (p < 0.05). Additionally, an effect of coherence was found for stimulus-aligned CMC when the RDM contained evidence for extension (panel B, F(34,1) = 5.53, p = 0.0229). No post-hoc tests were ran for this analysis.
